# Oral Verruciform Xanthoma: A Case Report and Literature Review

**DOI:** 10.1155/2013/528967

**Published:** 2013-12-16

**Authors:** Akshay Shetty, Kourosh Nakhaei, Yogesh Lakkashetty, Maryam Mohseni, Iman Mohebatzadeh

**Affiliations:** ^1^Department of Oral and Maxillofacial Surgery, Sri Rajiv Gandhi College of Dental Sciences and Hospital, Cholanagar, RT Nagar, Bangalore 560032, India; ^2^Sri Rajiv Gandhi College of Dental Sciences and Hospital, Cholanagar, RT Nagar, Bangalore 560032, India; ^3^Department of Oral and Maxillofacial Pathology, Sri Rajiv Gandhi College of Dental Sciences and Hospital, Cholanagar, RT Nagar, Bangalore 560032, India

## Abstract

Verruciform xanthoma is a benign mucocutaneous, uncommon, nonsymptomatic lesion of uncertain etiopathology, which occurs mostly on the oral mucosa of middle-aged individuals. Histopathologically, VX is diagnosed by presence of lipid-laden foam cells in papillary region of connective tissue. A 60-year-old male patient presented with a painless growth on the left buccal mucosa. On clinical examination a yellowish white exophytic lesion, measuring 11 × 7 mm in size, was found, which was cauliflower-shaped on inspection and painless on palpation. Histopathological examination revealed varying degrees of surface parakeratosis and the accumulation of numerous foam cells in the connective tissue papillae among the uniformly elongated epithelial ridges. On immunohistochemical staining, there was a neutrophilic infiltrate of the epidermis with CD68 positive xanthoma cells restricted to the papillary dermis, mixed with other chronic inflammatory cells.

## 1. Introduction

Verruciform xanthoma (VX) is an uncommon benign mucocutaneous lesion that resembles virus-induced papilloma but has an unknown etiology and uncertain nature. It was first reported by Shafer in 1971. VX usually occurs in the oral mucosa of the middle-aged individuals. It most commonly presents with a verrucous appearance. However in some instances it may appear polypoid, papillomatous, or sessile. It occurs as a small (0.2–2 cm), solitary, asymptomatic, slow growing, white or yellowish red lesion with no sex predilection [1, 2].

Histologically VX is distinguished from other lesions by the presence of large numbers of foam cells in, and essentially limited to, the connective tissue papillae. The foam cells on ultrastructural studies have been concluded to be fat-laden macrophages [3, 4]. Other cell types, including Langerhans cells, intraepithelial neutrophilic infiltrate, and even fibroblasts, have been reported [1–3]. A variable degree of parakeratosis is observed which is present in the crypts between papillae which are of variable length and thickness, often extending close to the surface. The rete pegs are extremely elongated and uniform [2]. Almost every VX case is diagnosed on histological examination as the clinical appearance is not diagnostic. Differentiation from other lesions with foamy or granular cells is not difficult as the VX is the only lesion to have these cells confined to the papillae [4, 5].

Differential diagnosis includes erythroplasia of Queyrat (Bowen disease of the glans penis), seborrheic keratosis, verrucous carcinoma, verruca simplex, and condyloma acuminatum [6, 7]. The treatment of the VX lesion involves local surgical excision which is almost always curative, and recurrence is rare [2].

## 2. Case Presentation

**Figure 1:**
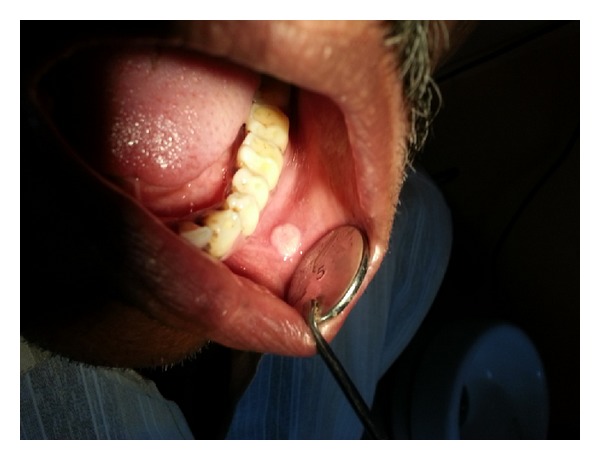
Clinical photograph showing the exophytic lesion on the left buccal mucosa.

A-60-year old male patient presented with the chief complaint of a painless growth on the left buccal mucosa (Figure 1). He had a habit of smoking for the past 10 years. On clinical examination, a yellowish white exophytic lesion, measuring 11 × 7 mm in size, was found, which had a cauliflower shape on inspection. The lesion was asymptomatic and soft in consistency. Lymph nodes were not palpable. There were no systemic diseases and he was otherwise healthy. A provisional diagnosis of papilloma was made. After clinical examination an excisional biopsy was taken to rule out malignancy.

**Figure 2:**
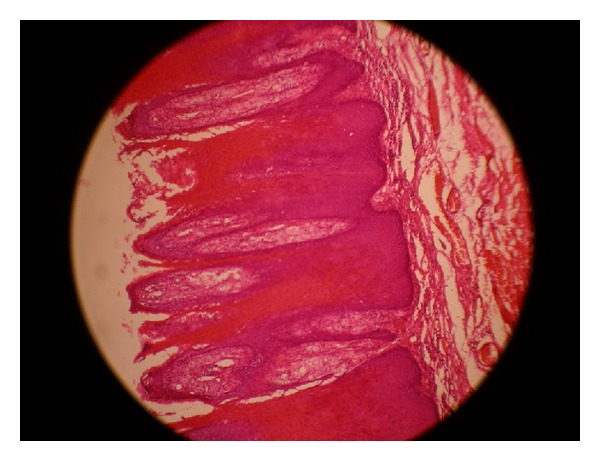
Showing epithelial hyperplasia with parakeratosis and elongated rete pegs. There is an abundance of foam cells in the connective tissue papilla (H&E stain 10x).

**Figure 3:**
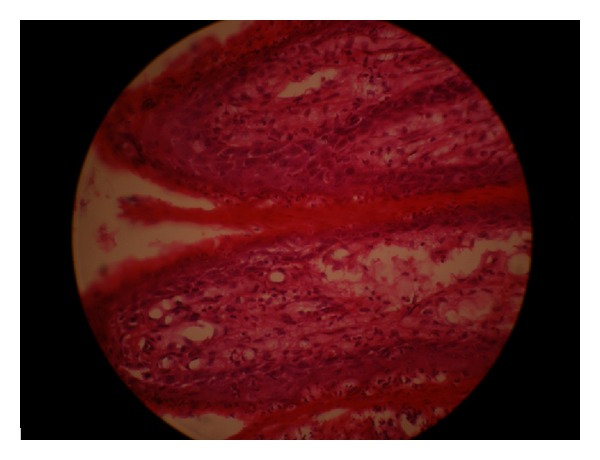
Presence of large numbers of lipid-laden foamy histiocytes confined to the connective tissue papillae (H&E stain 40x).

On histopathological examination, the hematoxylin and eosin (H&E) stained sections showed parakeratotic epithelium with columns of parakeratin plugging into it. Owing to the uniformly elongated rete pegs, deep connective tissue papillae were seen, some of which extended into overlying surface epithelium (Figures 2 and 3). Few neutrophils were also seen in the upper spinous layer. The papillary zone of lamina propria showed numerous lipid-laden foam cells. There was no evidence of dysplasia or malignancy. The fact that the foam cells were confined to the papillary region of connective tissue confirmed the diagnosis as VX.

**Figure 4:**
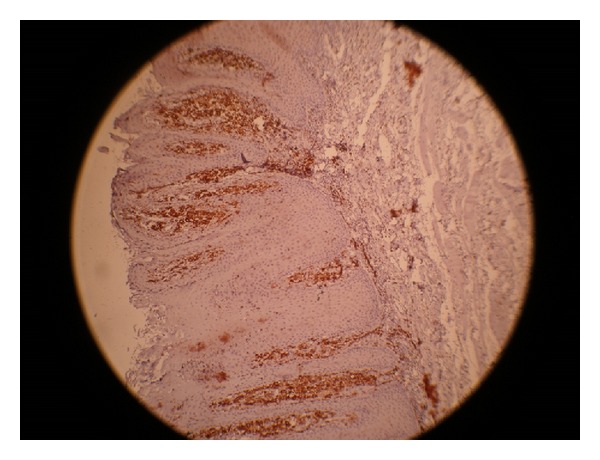
Showing foam cells with strong cytoplasmic CD68 immunostaining. The epithelial cells were negative (IHC stain 10x).

**Figure 5:**
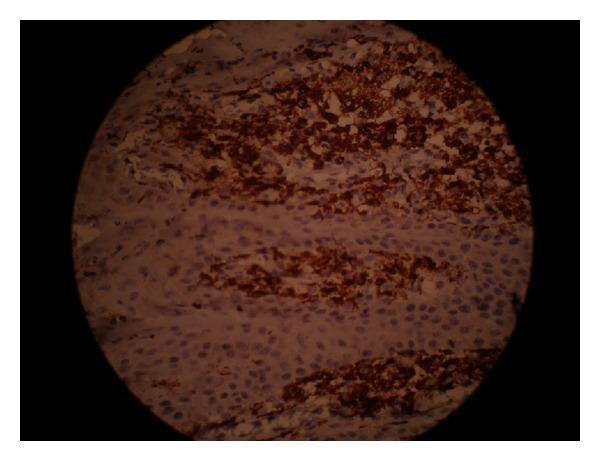
The infiltrated foam cells in the papillary dermis were CD68 positive (IHC stain 40x).

The immunohistochemical staining for CD68 was positive for the foamy macrophages. All the foam cells were strongly stained with anti-macrophage antibodies (Figures 4 and 5).

**Figure 6:**
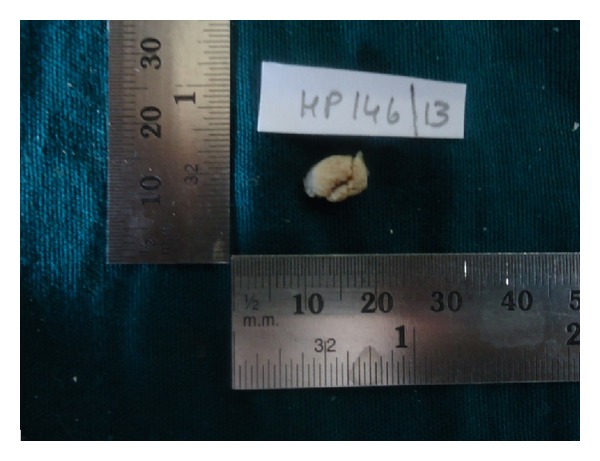
Showing the lesion after excision.

The treatment was performed under local anesthesia. The whole lesion was excised in one piece during excisional biopsy (Figure 6). Postoperative check-up showed no sign of recurrence.

## 3. Discussion

Verruciform xanthoma is a rare lesion, accounting for 0.025–0.095% of all cases [3] with an unknown etiopathology. It can be because of the damage to the squamous cells due to trauma, irritation, or infection, which can cause increased epithelial turnover leading to the disease. The epithelial breakdown leads to an inflammatory response and a subsequent release of lipid material from the degenerated cells [2, 8–11]. Most of the cases occur in otherwise healthy individuals. Due to its clinical and histopathological resemblance to human papilloma virus-induced lesions, verruciform xanthoma was believed to be caused by HPV. However, most investigators have not found any evidence for the presence of HPV in these lesions [3]. However a few cases have been reported which were associated with inflammatory conditions such as pemphigus vulgaris, lichen planus, discoid lupus erythematosus, warty dyskeratoma, epidermal nevus/CHILD nevus, dystrophic epidermolysis bullosa, and seborrheic keratosis [12]. A few cases have also been reported to be associated with disorders of lipid metabolism [13]. On serological examination, our case had a normal serum lipid profile. The lesion is benign, nonsymptomatic, and slow growing and rarely exceeds 2 cm in size. It is sessile or pedunculated and can resemble leukoplakia or squamous papilloma. It occurs most commonly in 4th–6th decade of life with equal distribution between both sexes. However, it has been reported that there is a slight male predilection [1, 2, 14]. The lesion is usually present in the intraoral regions especially on alveolar ridge, gingiva, followed by buccal mucosa, palate, floor of the mouth, and lip. Extra oral sites include vulva, scrotum, penis, and skin of thigh and perineum, which are usually associated with other conditions like lymphedema, epidermal nevi, congenital hemidysplasia, and limb defect syndrome [6, 7, 15].

Histologically, VX shows three patterns: verrucous (most common), flat, and papillary (least common) [16, 17]. There is parakeratosis of the hyperplastic epithelium which is variable in extent and is usually more marked in the verrucous and papillary patterns. The rete pegs are elongated, uniform, and thin, with deep central keratinized clefts and keratin plugging. However, there is no evidence of dysplasia. The characteristic histological feature is the presence of xanthoma cells in the connective tissue [2, 18]. The lipid-laden foam cells are present in the superficial connective tissue. There is a controversy over the exact origin of these cells. They are said to be a lineage of monocytes/macrophages [19, 20]. The lipid found in the xanthoma cells is said to be the same as seen in other inflammatory reactions [12]. In our case, the foam cells showed strong CD68 immunoreactivity. CD68 is a cell marker confirming the possible role of macrophages in the formation of foam cells [12].

The treatment of choice is complete surgical excision which is very effective with no recurrence. However, a recurrent VX of the vulva has been reported in a 30-year-old woman, 8 years after the initial treatment [20]. Histopathological examination of the biopsies should be performed to distinguish VX from other verrucous lesions [2, 21].
